# Enterprise risk management implementation challenges in medical laboratories in Harare, Zimbabwe

**DOI:** 10.1002/hsr2.70088

**Published:** 2024-09-23

**Authors:** Donald Vhanda, Kudzai Chinowaita, Frank Chinowaita, Reynold Vhanda, Brenda Nherera, Tafadzwa Dzinamarira, Itai James Blessing Chitungo

**Affiliations:** ^1^ Chemical Pathology Unit, Department of Laboratory Diagnostic and Investigative Sciences, Faculty of Medicine and Health Sciences University of Zimbabwe Zimbabwe; ^2^ Graduate Business School Bindura University of Science Education Zimbabwe; ^3^ Department of Actuarial Sciences National University of Science and Technology Zimbabwe; ^4^ Department of Pathology (Microbiology) Midlands State University Zimbabwe; ^5^ Department of Marketing National University of Science and Technology Zimbabwe; ^6^ ICAP at Columbia University Harare Zimbabwe

**Keywords:** healthcare, implementation challenges, medical laboratory, risk management

## Abstract

**Background:**

Medical laboratories play crucial roles in healthcare, and effective enterprise risk management (ERM) is necessary to ensure business continuity, patient safety, and quality of care. In medical laboratories, ERM is important for enhancing patient safety, regulatory compliance and accreditation, quality management, business continuity, and cyber security. By following ERM principles and approaches, the medical laboratories can proactively manage their risks, improve patient safety, and maintain a high level of quality and reliability of outcomes. However, implementing ERM in medical laboratories faces unique challenges. This study explored the specific challenges and offers practical solutions for overcoming them to ensure successful ERM implementation in Harare, Zimbabwe.

**Methodology:**

A cross‐sectional survey was done through 41 self‐administered questionnaires and interviews with medical laboratory staff from the six main medical laboratories in Harare. Data were analyzed using the Statistical Package for Social Sciences version 22. Quantitative data were analyzed using descriptive statistics such as frequencies and percentages. Qualitative data were analyzed using mean scores of Likert scale responses.

**Results:**

The main challenges identified in the study included increased workload, staffing, organizational structures, timeliness of information, inadequate information technology support, and insufficient financial support in ERM. These can be addressed by portfolio management of risks, leveraging on cutting edge technology, restructuring to ensure swift responses to issues and redistributing staff workload, and training personnel to avoid burnouts and ensure maximum efficiency.

**Conclusion:**

Implementing ERM in medical laboratories requires understanding and addressing these challenges. By following the ERM principles and approaches the medical laboratories can proactively manage their risks, improve patient safety and maintain a high level of quality and reliability of outcomes ERM is still a new approach in the sector and needs further research.

## 1 BACKGROUND

The medical laboratory sector plays a crucial role in healthcare management globally, with over 70% of medical decisions based on medical laboratory testing.^1^ However, medical laboratories operate in complex and dynamic environments full of risks that can impact business continuity, patient safety, and ultimately quality of care.^2^, ^3^, ^4^ Therefore, effective risk management (RM) approaches are pivotal in enhancing high levels of reliability of outcomes and improving patient safety and user experiences as alluded to in the International Organization for Standardization (ISO) 15189 medical laboratories standard.^3^, ^4^, ^5^ The escalating use of novel approaches in the diagnostic landscape calls for the use of advanced risk assessment tools to safeguard the human health and the environment.

RM is the broad subject that refers to the systematic process of identifying, assessing, and mitigating for potential risks.^6^ It can be broken down to clinical risk management if it focuses on patient safety and quality of care, and it can be referred to as enterprise risk management (ERM) if it is a comprehensive approach that considers all types of risks across the entire organization, in a holistic manner.^3^, ^5^, ^6^ The traditional silo approach to RM has given way to newer ERM approaches in various sectors. Thus, ERM has emerged as a powerful tool in systematically identifying, preventing as well as mitigating these risks that threaten organizational performances and accreditation.^7^ Nevertheless, the concept of ERM brings with it a fair share of implementation challenges within the medical laboratory sector.

ERM is the holistic or integrated management of risks facing an organization.^8^, ^9^ As a process, ERM is affected by the organization's stakeholders, particularly the board, management, and personnel involved in strategy setting, risk identification, and mitigation. The approach was born out of the realization of complexities, dynamism, and rapid changes facing institutions in the modern era.^5^, ^8^, ^10^ Therefore, ERM is a systematically integrated and disciplined approach to managing risks within companies to ensure they achieve set goals and unlock value for their stakeholders. Whereas the traditional RM approach took a silo approach to individual risk type ERM uses a portfolio point of view, of identifying risk, interdependencies, and interrelationships. It offers the ability to manage risks within and across business units and improves an organization's ability to identify and seize opportunities, thereby giving an organization a competitive edge in the market and a proactive approach to handling risky events. RM involves anticipation of what could happen, the assessment of the frequency of these errors as well as the consequences or the severity of the effects caused by it, and finally to decide what can be done to reduce the risk to a tolerable or an acceptable level.^11^ In medical laboratories, ERM is important for enhancing patient safety, regulatory compliance and accreditation, quality management, business continuity, and cyber security. By following ERM principles and approaches, the sector can proactively manage their risks, improve patient safety, and maintain a high level of quality and reliability of outcomes.^2^, ^3^, ^12^ ERM can promote efficiency, effectiveness, and responsiveness while mitigating risks and optimizing organizational performance in delivering health services and laboratory operations.^13^

Kilbridge and Classen (2008) reiterated that today's healthcare environment is characterized by emerging and re‐emerging risks, diverse organizations, continuously changing operational codes, financial rules, ever‐changing and increasing regulations, information symmetry with consumers, and technological advancements.^2^ Several studies have been done on RM implementation challenges and practices in multiple organizations and sectors.^14^, ^15^, ^16^, ^17^ The results were varied practices and also different implementation challenges depending on the setting. The majority were done in developed countries like the United States of America (USA), Europe, and a few in Africa. Some of the implementation challenges highlighted included a lack of ERM culture within organizations, lack of expertize, resource constraints, technological barriers, regulatory complexities, lack of top management support, and insufficient financial backing, among others.^8^, ^15^, ^18^ The medical laboratories sectors' highly specialized and constantly evolving nature further exacerbates these challenges, making it important to explore the context‐specific situations so that innovative solutions can be proffered.

This manuscript aims to interrogate and explore the specific challenges faced by medical laboratories in Harare, Zimbabwe, in implementing ERM and to identify solutions and strategies to overcome them. By interrogating the complexities in this sector, we hope to provide valuable insights and guidance for policymakers, regulators, managers, and the laboratory professionals at large seeking to strengthen their RM capabilities, push for ISO 15189 accreditation, improve stakeholder value, and deliver high‐quality patient care.

## 2 METHODS

### 2.1 Study design and study population

In May 2018, we performed a cross‐sectional study through 41 self‐administered questionnaires and interviews with the medical laboratory staff from the six main medical laboratories in Harare, Zimbabwe. The study took a survey approach to investigate the ERM implementation challenges faced by medical laboratories.

Sampling was done in two stages. We first purposively targeted six big laboratories in Harare, where most of the laboratories' head offices are concentrated. Purposive sampling approach was chosen to increase the chances of getting participant laboratories that met the inclusion criteria detailed below. Second, a stratified random sampling technique was applied to select participants within the organization. Staff at various levels within the organization (management, practitioners, front officers, nursing department, as well as the housekeeping departments) formed the different strata. Different strata within organizations were assessed to check if there was information symmetry within the organizations because that is pivotal for successful ERM implementation. Random sampling was used in the second stage to reduce bias associated with the study participants' selection. The randomly selected participants were then given questionnaires and interviewed to assess the ERM implementation challenges in the laboratory sector. To avoid ambiguity and improve the reliability of the questionnaire and interview guide, the tools were pilot‐tested before being administered to the actual study participants.

Self‐administered questionnaires and interviews were adopted because these are data collection approaches that allow researchers to collect in depth and contextualized data from a diverse sample in a timely, efficient, and cost‐effective manner. Self‐administering also helps to achieve a higher return rate and also offers the research more leeway to explain the questions to the study participants, hence reducing information bias.

### 2.2 Inclusion criteria

Employees from the six medical laboratory organizations based in Harare, who consented to participate in the study were included in the study. The targeted laboratories were either accredited or actively working towards accreditation, as these had the biggest likelihood of implementing RM as stipulated in the ISO 15189 standard.

### 2.3 Exclusion criteria

Employees who refused to participate in the study were excluded. Participants who did not belong to the six big laboratories were not included in the study.

### 2.4 Sample size

The case studies (sampled population) gave pointers to areas that can be explored further in bigger studies. Hurdles to effective ERM implementation were interrograted and identified in this exploratory study and shed more light on areas that can be investigated further, in efforts to reduce risks to tolerable or acceptable levels. Beasley and Branson (2005) argue that “large organizations are more likely to report complete ERM processes.”^19^ Hence, these were used as case studies upon which the ERM implementation challenges were investigated.

To come up with a sample size (*N*), a Raosoft formula was used

$$\frac{Z^{2}\times (p)\times (1-p)}{C^{2}},$$

where *Z* = level of confidence (95%)

*p* = percentage picking choice expressed as a decimal

*C* = margin of error.^20^

### 2.5 Data analysis

Data was analyzed using the Statistical Package for Social Sciences (SPSS) version 22 (SPSS Inc.). Quantitative data were analyzed using descriptive statistics, frequencies and percentages. For qualitative data, the overall ERM score for the organization was obtained by averaging Likert scale scores. The mean scores were extracted from the SPSS version 22. An individual dimension score was computed using a similar procedure of corresponding sub‐dimensions. The mean scores were used to determine ERM implementation challenges, using descriptive analysis. Rechecking the questionnaires ensured that biases in question formulations were eliminated. Reliability was analyzed using the Cronbach's alpha on the SPSS.

### 2.6 Ethics approval and consent to participate

All methods were carried out according to the relevant guidelines and regulations for carrying out cross‐sectional studies using questionnaires and interviews. The ethical approvals were obtained from the Bindura University of Science Education Ethics and Bio‐Safety Research Committee. Approvals were obtained from the participating organizations to interview their staff members and administer questionnaires. Informed consent was obtained from all participants before enrolling in the study. Participants were also free to withdraw at any time during the course of the questionnaire administration and interview.

## 3 RESULTS

A total of 50 questionnaires were delivered, and 41 were returned, representing an 82% response rate, which is satisfactory for the generalization of results. The high response rate was achieved due to the use of the purposive sampling technique that was adopted in this research. Males constituted 61.6% of the sample.

The data collected passed the reliability and consistency test as depicted by Cronbach's alpha of 0.748 for all the factor dimensions, demonstrating a high level of consistency and reliability.

### 3.1 Age distribution

**Figure 1:**
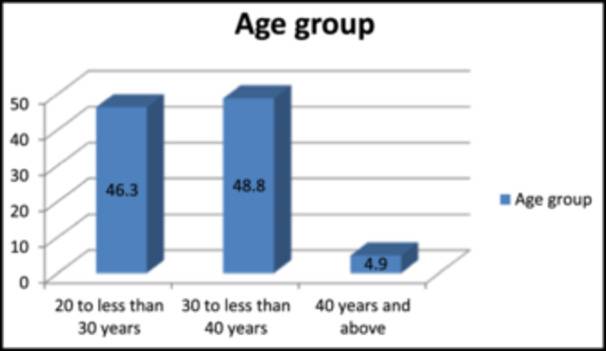
Percentage distribution of respondents by age.

The age distribution is shown in Figure 1 below.

### 3.2 Work experience of the respondents

The majority of the respondents 21 (51.2%) were in the less than 5 years category of experience. Nineteen (46.3%) were in the experienced category of 5–10 years, and only 1 (2.5%) was in the 10–15 years category.

### 3.3 ERM implementation challenges

Several challenges were highlighted by the respondents in Table 1 below. The following are the challenges that stood out as being apparent in the industry.

**TABLE 1:** Summary of the ERM implementation challenges and their respective mean scores.

ERM implementation challenges	Mean score (range 1–5)
Over‐regulation	2.6585
Lack of support from senior management.	2.9756
Lack of information needed	3.1000
Failure by leaders to spearhead the project	3.1463
Lack of RM expertize within the organization.	3.1707
Lack of financial resources	3.3415
Insufficient investment for ERM implementation	3.3500
The wide discrepancy between expectations and practices in RM implementation	3.4146
Inadequate technology support	3.5000
Timeliness of information	3.5128
Organization structure deters RM implementation	3.5500
Staffing	3.6829
Increased workload	4.0732

*Note*: The following scales are used to measure the importance of respondents' perceptions of the ERM implementation challenges. Mean scores ranging from 1.0 ≤ M < 1.8: Very low importance.

■ Mean scores ranging from 1.8 ≤ M < 2.6: Low Importance.

■ Mean scores ranging from 2.6 ≤ M ≤ 3.4: Neutral.

■ Mean scores ranging from 3.4 < M ≤ 4.2: High Importance.

■ Mean scores ranging from 4.2 < M ≤ 5.0: Very high importance.

### 3.4 Results interpretation

ERM challenges that scored above 3.4 were of high importance in the sector. These were increased workload, staffing, organizational structures, timeliness of information, inadequate information technology support, and insufficient financial support in ERM.

Increased workload (mean 4.0) can lead to distraction, burnout, corner‐cutting, and inadequate reviews. Organizations need to prioritize maintaining a balanced workload, and sufficient allocation of resources to ensure effective RM. Closely related to increased workload is staffing (mean 3.7). Inadequate staffing results in insufficient skills, high turnover, inadequate resources, and overreliance on a few individuals. Organizations need to ensure adequate staffing, proper training, and development, as well as having replacement policies for the successful construction of systems that can effectively implement ERM. Huge workloads and understaffing are synonymous challenges as organizations try to manage costs, especially in third‐world countries like Zimbabwe.

Organization structure (mean score 3.6) is crucial for ERM implementation. Clear cut structures ensure efficient communication, defined roles and responsibilities scalability of ERM efforts. Therefore organizations must adapt their structures to support ERM implementation. ERM is a new concept to the sector, and most organizations have not adapted to match the new demands.

Timeliness of information (mean score 3.5) has huge impact on ERM implementation. It ensures timely responsiveness, proactive approach to problem solving, and real time risk identification, assessment, and mitigation. Most big organizations are tangled in the bureaucratic approaches to doing business, hence delays in responding to raised challenges.

Inadequate technological support (mean score 3.5) can also be a huge impediment to effective ERM implementation. It leads to insufficient operations, poor data collection, poor reporting, more manpower requirements, more repeat services, more security risks, and ultimately more costs to the organizations. Investing in cutting‐edge technological tools gives organizations competitive advantages. Lack of financial muscles handicaps investment in modern technologies in third‐world countries.

ERM approaches look at the interdependence and interconnectedness of the whole organization holistically; therefore, it offers possibilities of finding lasting solutions to these challenges more cost‐effectively.

## 4 DISCUSSION

The various challenges that affected the medical laboratory sector during the survey are highlighted in Table 1 and interpreted in the section above. The main challenges identified in the study included increased workload, staffing, organizational structures, timeliness of information, inadequate information technology support, and insufficient financial support in ERM.

### 4.1 Increased workload

Increased workload was reportedly one of the key challenges, as illustrated by a high mean score of 4.07 on a Likert scale of 1–5. The majority of the participants indicated that the ERM implementation was associated with more paperwork. One of the respondents stated, “*We are already overloaded with the quality systems and accreditation requirements so the burden is too much*.” All of the laboratories in the study were either accredited or were working towards accreditation for ISO 15189, and the Zimbabwe National Certification (ZNC) program. That might have had a bearing on the viewpoint on workload. However, with the liquidity crunch prevailing in Zimbabwe, staff turnover is high as companies try to reduce their operational costs.^21^ Leggio (2006) also cited increased workload as one of the major challenges of ERM implementation.^22^ Corporate culture was reported as the biggest challenge to ERM implementation in other studies.^15^, ^18^

### 4.2 Staffing challenges

The respondents posited that the staffing issue was one of the major challenges in ERM implementation as exhibited by a mean score of 3.68. This challenge is closely related to increased workload. Staffing issues ranged from inadequacy, incompetency, and wrong culture. Some respondents asked, “*Where do we get the training for those risk management issues you are asking us about?*” Any sound business strategy should be backed by the proper staffing or structure that matches what needs to be achieved. That is the reason why this particular factor was assessed. Most laboratories in Zimbabwe have skeletal staff due to the harsh economic environment prevailing in the country. The finding concurs with Rostami et al. (2015) and Yaraghi's (2011) findings that human resources issues are some of the challenges faced in ERM implementation in their respective studies.^23^, ^24^ In a study carried out in the United Kingdom construction industry, lack of experienced personnel and high staff turnover were also highlighted as key ERM implementation challenges.^23^

### 4.3 Organizational structure

Successful strategy implementation hinges on a sound organizational structure. The majority of the organizations indicated that they do not have risk departments, officers, or committees. Study participants' responses showed that organizational and reporting structures were key constraints in ERM implementation. An overall mean score of 3.55 was attained. Most respondents were persuaded by the fact that RM was easier managed with a responsible department overlooking the day‐to‐day RM activities in the organization. It was also found that only one organization had an RM department in the laboratories recruited in the study. Others were incorporating RM issues into the quality assurance department but even the quality managers felt that roles needed separation. Other studies also indicated that organizational structure was a crucial challenge in RM implementation.^25^, ^26^ Several scholars agree that having a central risk function along with risk policies, and objectives is a pivotal step in RM implementation.^18^, ^27^ Nocco et al. (2006) also pointed out that senior‐level management buy‐in is a key ingredient of the effective implementation of ERM.^28^

### 4.4 Timeliness of information

Failure to respond timely to raised issues is the major weakness of many organizations as far as ERM implementation is concerned.^29^ The mean score was 3.51. Financial issues and bureaucracy were implicated as the main reason behind late or no response. Interview follow‐ups showed that most organizations were faced with delays in addressing the raised concerns. One employee remarked,“*We always raise these issues in our reports and meetings but the management pays a deaf ear, if you follow up with the accounts department they tell you we don't have money*.” Other studies also found the time factor to be a hindrance to RM implementation.^15^, ^17^ Timeliness of information dissemination and addressing the raised issues can be a key impediment in resource‐limited settings and institutions with a lot of bureaucracy.

### 4.5 Inadequate technological support

Lack of technological support was found to be a hindrance to ERM implementation with a mean score of 3.50. RM thrives on efficient technological infrastructure for reporting purposes. This might be caused by limited financial resources in the industry due to the liquidity crunch in the country. At one government laboratory, the employees laughed off the suggestions of the risk dashboard in the interview, “*most of the time we enter results manually because the system is always down, our machines are not even interfaced yet you are talking about dashboards? We would want that but we are still far off at the moment*.” Technological developments in equipment and laboratory information systems are key to achieving objectives. Risk dashboards, risk heat maps, and risk models are important in the implementation of ERM.^10^, ^25^, ^30^ Other studies also alluded to technological challenges being one of the drawbacks to ERM implementation.^2^, ^30^

The limitations of this study include that self‐administered questionnaires and interviews could have resulted in some bias in the study. Limited resources also led the researchers to adopt a sampling approach. It would have been more representative to increase the sample size or possibly sample every laboratory in Harare and conduct more in‐depth interviews to gain more understanding of the challenges faced by the respective organizations.

Nevertheless, the study being the first of its kind in the particular study population, can offer helpful insights that can be expanded on in future studies. The authors recommend bigger studies, which are more representative, using triangulation of various methods to generate more insights into ERM in the medical laboratories sector.

### 4.6 Recommendations

Organizations can benefit more from adopting management approaches that properly forecast their staff requirements and workload distribution to avoid staff burnout. Adopting culture change programs that focus more on responding to staff and patient needs as highlighted in the new ISO 15189:2022 standard, and cut on bureaucracy is key. Swift responses to raised complains are the hallmark of successful organizations. Investing in technological advancements on both the operational (cutting edge technology) and management (use of risk dashboards) perspective enhances organizational efficiency and cuts on staffing requirements. Policymakers and regulatory authorities like the Medical Laboratory and Clinical Scientists Council of Zimbabwe (MLCSCZ) can prioritize the above to improve the push towards accreditation and revamping the quality of care in medical laboratories in Zimbabwe.

### 4.7 Further research

Areas of further research could include exploring effective ERM strategies, evaluating the long‐term impact of ERM implementation, or assessing the role of technological advancements in ERM implementation and also testing the identified challenges in a bigger sample.

## 5 CONCLUSIONS

Implementing ERM in medical laboratories requires understanding and addressing these challenges. The ERM implementation challenges faced Zimbabwe laboratory sector included increased workload, staffing issues, improper organizational structures to support ERM implementation, delays in intervention or response to raised issues, and inadequate technology to track key performance indicators and risk dashboards. By following the ERM principles and approaches, the medical laboratories can proactively manage their risks, improve patient safety, and maintain a high level of quality and reliability of outcomes. That ensures regulatory compliance and satisfies the requirements of accreditation bodies. ERM is still a new approach in the sector and needs further research.

## AUTHOR CONTRIBUTIONS

**Donald Vhanda**: Conceptualization; investigation; funding acquisition; writing—original draft; writing—review and editing; validation; methodology; resources; supervision; data curation; visualization; project administration. **Kudzai Chinowaita**: Methodology; validation; visualization; writing—review and editing; software; formal analysis. **Frank Chinowaita**: Writing—review and editing; methodology; formal analysis. **Reynold Vhanda**: Methodology; software; formal analysis; validation; writing—review and editing. **Brenda Nherera**: Writing—review and editing; validation; visualization. **Tafadzwa Dzinamarira**: Writing—review and editing; validation; visualization. **Itai James Blessing Chitungo**: Writing—review and editing; visualization; validation.

## CONFLICT OF INTEREST STATEMENT

The authors declare no conflicts of interest.

## TRANSPARENCY STATEMENT

The lead author Donald Vhanda affirms that this manuscript is an honest, accurate, and transparent account of the study being reported; that no important aspects of the study have been omitted; and that any discrepancies from the study as planned (and, if relevant, registered) have been explained.

## Data Availability

The data that support the findings of this study are available from the corresponding author upon reasonable request.

## References

[1] Sikaris KA . Enhancing the clinical value of medical laboratory testing. Clin Biochem Rev. 2017;38(3):107‐114. 29332975 PMC5759162

[2] Kilbridge PM , Classen DC . The informatics opportunities at the intersection of patient safety and clinical informatics. J Am Med Inform Assoc. 2008;15(4):397‐407. 18436896 10.1197/jamia.M2735 PMC2442268

[3] Njoroge SW , Nichols JH . Risk management in the clinical laboratory. Ann Lab Med. 2014;34(4):274‐278. 24982831 10.3343/alm.2014.34.4.274 PMC4071183

[4] Guzel O , Guner EI . ISO 15189 accreditation: requirements for quality and competence of medical laboratories, experience of a laboratory I. Clin Biochem. 2009;42(4):274‐278. 19863920 10.1016/j.clinbiochem.2008.09.011

[5] Elamir H . Enterprise risk management and bow ties: going beyond patient safety. Business Process Manag J. 2019;26:770‐785.

[6] Gjerdrum D , Peter M . The new international standard on the practice of risk management—a comparison of ISO 31000: 2009 and the COSO ERM framework. Risk Manage. 2011;31(21):8‐12.

[7] Singh AV , Varma M , Rai M , et al. Advancing predictive risk assessment of chemicals via integrating machine learning, computational modeling, and chemical/nano‐quantitative structure‐activity relationship approaches. Adv Intell Syst. 2024;6(4):1‐15.

[8] Kanhai C , Ganesh L . Factors influencing the adoption of enterprise risk management (ERM) practices by banks in Zimbabwe. Int J Bus Commer. 2014;3(6):01‐17.

[9] Liebenberg AP , Hoyt RE . The determinants of enterprise risk management: evidence from the appointment of chief risk officers. Risk Manage Insurance Rev. 2003;6(1):37‐52.

[10] Razali AR , Tahir IM . Review of the literature on enterprise risk management. Business Manag Dyn. 2011;1(5):8‐16.

[11] Singh AV , Shelar A , Rai M , et al. Harmonization risks and rewards: nano‐QSAR for agricultural nanomaterials. J Agric Food Chem. 2024;72(6):2835‐2852. 38315814 10.1021/acs.jafc.3c06466

[12] Crickette G , Drobnis K , Egerdahl R , et al. An Overview of Widely Used Risk Management Standards and Guidelines. Risk Insur Manag Soc Inc; 2011.

[13] Singh AV , Varma M , Laux P , et al. Artificial intelligence and machine learning disciplines with the potential to improve the nanotoxicology and nanomedicine fields: a comprehensive review. Arch Toxicol. 2023;97(4):963‐979. 36878992 10.1007/s00204-023-03471-x PMC10025217

[14] Dabari IJ , Saidin SZ . A theoretical framework on the level of risk management implementation in the Nigerian banking sector: the moderating effect of top management support. Proc ‐ Soc Behav Sci. 2014;164:627‐634.

[15] Prioteasa AL , Ciocoiu CN . Challenges in implementing risk management: a review of the literature. Proceedings of the International Management Conference. Faculty of Management, Academy of Economic Studies, Bucharest, Romania; 2017. p. 972‐980.

[16] Beasley MS , Branson B , Hancock B . The state of risk oversight: an overview of enterprise risk management practices. 13^th^ ed. ERM Poole Coll Manag N C State Univ; 2022.

[17] Acharyya M , Mutenga S . The benefits of implementing enterprise risk management: evidence from the non‐life insurance industry. Enterp Risk Manag. 2013;6(1):22‐24.

[18] Fraser JRS , Simkins BJ . The challenges of and solutions for implementing enterprise risk management. Bus Horiz. 2016;59(6):689‐698.

[19] Beasley MS , Clune R , Hermanson DR . Enterprise risk management: an empirical analysis of factors associated with the extent of implementation. J Account Public Policy. 2005;24(6):521‐531.

[20] Hightower C , Scott K . Infer more, describe less: more powerful survey conclusions through easy inferential tests. Issues Sci Technol Librariansh. 2012;69(Spring):1‐23.

[21] Chimbari P . Public sector corporate governance in Zimbabwe: the nexus between the ZIMCODE and state‐owned enterprises. Int J Econ Commer Manag. 2017;5(7):212‐221.

[22] Leggio KB . Managing Enterprise Risk: What the Electric Industry Experience Implies For Contemporary Business. Elsevier; 2006.

[23] Rostami A , Sommerville J , Wong IL , Lee C . Risk management implementation in small and medium enterprises in the UK construction industry. Eng, Constr Archit Manag. 2015;22(1):91‐107.

[24] Yaraghi N , Langhe RG . Critical success factors for risk management systems. J Risk Res. 2011;14(5):551‐581.

[25] Acharyya M . An empirical study on enterprise risk management in insurance. In: Olson DL , Wu D , eds. New Frontiers in Enterprise Risk Management. Springer; 2008:39‐55.

[26] Vila LL , Buccellato V . Implementation of a sustainable enterprise risk management framework: the administrator on duty model. J Hosp Adm. 2016;5(2):80.

[27] Kerstin D , Simone O , Nicole Z , Lehner OM . Challenges in implementing enterprise risk management. ACRN J Finance Risk Perspect. 2014;3(3):14.

[28] Nocco BW , Stulz RM . Enterprise risk management: theory and practice. J Appl Corporate Finance. 2006;18(4):8‐20.

[29] Singh AV , Bansod G , Mahajan M , et al. Digital transformation in toxicology: improving communication and efficiency in risk assessment. ACS Omega. 2023;8(24):21377‐21390. 37360489 10.1021/acsomega.3c00596 PMC10286258

[30] Etges APBS , de Souza JS , Kliemann Neto FJ , Felix EA . A proposed enterprise risk management model for health organizations. J Risk Res. 2019;22(4):513‐531.

